# Feature Selection for Machine Learning Based Step Length Estimation Algorithms

**DOI:** 10.3390/s20030778

**Published:** 2020-01-31

**Authors:** Stef Vandermeeren, Herwig Bruneel, Heidi Steendam

**Affiliations:** Department of Telecommunications and Information Processing–IMEC, Ghent University, 9000 Gent, Belgium; herwig.bruneel@ugent.be (H.B.); Heidi.Steendam@UGent.be (H.S.)

**Keywords:** machine learning, feature selection, IMU

## Abstract

An accurate step length estimation can provide valuable information to different applications such as indoor positioning systems or it can be helpful when analyzing the gait of a user, which can then be used to detect various gait impairments that lead to a reduced step length (caused by e.g., Parkinson’s disease or multiple sclerosis). In this paper, we focus on the estimation of the step length using machine learning techniques that could be used in an indoor positioning system. Previous step length algorithms tried to model the length of a step based on measurements from the accelerometer and some tuneable (user-specific) parameters. Machine-learning-based step length estimation algorithms eliminate these parameters to be tuned. Instead, to adapt these algorithms to different users, it suffices to provide examples of the length of multiple steps for different persons to the machine learning algorithm, so that in the training phase the algorithm can learn to predict the step length for different users. Until now, these machine learning algorithms were trained with features that were chosen intuitively. In this paper, we consider a systematic feature selection algorithm to be able to determine the features from a large collection of features, resulting in the best performance. This resulted in a step length estimator with a mean absolute error of 3.48 cm for a known test person and 4.19 cm for an unknown test person, while current state-of-the-art machine-learning-based step length estimators resulted in a mean absolute error of 4.94 cm and 6.27 cm for respectively a known and unknown test person.

## 1. Introduction

The inclusion of multiple sensors in smartphones allowed us to develop new applications for these devices that enable us to monitor the physical activity and detect certain health issues of the user. Especially the inertial measurement unit (IMU), which is a combination of two or three sensors (an accelerometer, gyroscope and sometimes a magnetometer), is very useful for these kinds of applications. The IMU is able to measure the motion and orientation of a mobile device. Therefore, it can be used to count the number of steps a user takes, determine the traveled distance, track the location of the user, and even detect gait impairments [1,2]. In this paper, we will use the IMU, and more specifically the accelerometer, to determine the length of the steps a user takes.

In the literature, several approaches can be found to estimate the step length from IMU data. In the first class of approaches, i.e., the parametric based approaches [3,4,5,6], the step length is written as a function of different variables that are determined from the acceleration and sometimes the gyroscope data, and some parameters that need to be tuned to a specific user. In [3] the authors write the step length as a function of the maximum and minimum value of the vertical acceleration. In [4] the step length is modeled as a function of the sum of the absolute values of the acceleration magnitude. Further, in [5] the step length is taken proportional to the height of the user multiplied with the square root of the step frequency, and in [6] the authors model the step length as a function of the height of the user multiplied with a linear function of the step frequency. Common to all approaches is the presence of one or more parameters to be tuned.

In the second class of approaches, i.e., the Kalman filter-based methods, a Kalman filter is used to estimate the step length from a double integration of the acceleration in a fixed reference frame. An advantage of this approach, compared to the parametric based methods, is that no parameters need to be tuned to a specific user, while a disadvantage is that now both data from the accelerometer and gyroscope are needed. For example, in [7,8] the authors present a zero-velocity-update gait analysis system based on a Kalman filter for an IMU that is attached to the foot. The drawback of this approach is that the sensor is attached to the foot, which requires a dedicated battery-powered device in contrast to handheld solutions where a smartphone can be used. Another disadvantage of Kalman-based approaches is that the sensor measurements contain a bias, which will result in erroneous step length estimations.

The last approach uses supervised learning algorithms. In supervised learning, the goal is to model a function f(x) that transforms the input *x* into a wanted output *y* as accurately as possible. To determine this function, we provide the supervised learning algorithm with some labeled examples (the training set), i.e., for a known input *x* we also give the expected output *y* to be returned by the algorithm. After the training phase, we can use the supervised learning model to predict the output corresponding with the new input data. In the supervised learning approach for step length estimation—a regression problem—the step length estimation algorithm tries to learn the step length, based on examples from a training set. To this end, the algorithm extracts features, i.e., scalar values, from the measured data. Afterwards, this algorithm can use the model it learned from this training set to predict the step length for new data. In [9] the authors use a neural network to predict the step length based on features derived from a helmet-mounted accelerometer. Only four features are considered in this work, i.e., the maximal value, the minimal value, the variance and the integral of the acceleration magnitude from a step. In [10] the authors also use a neural network to predict the step length but this time with a foot-mounted accelerometer. In this work, five features are used, i.e., the mean stride frequency, the maximal acceleration, the standard deviation of the acceleration, the mean acceleration and the height of the test person. To evaluate the performance of the step length estimation, the step length was averaged over an entire walk, i.e., only the total traveled distance is determined and not the length of each individual step. If we want to track users with a People Dead Reckoning (PDR) approach, the length of each step separately, and not only the length of a sequence of steps, is needed because each step can have a different direction. In [11] the authors use the model of [3] to predict the step length, but instead of manually tuning the parameters to a specific user, the authors use a neural network to estimate the parameter based on the stride frequency and the height of the test person. In [12] the authors propose a deep learning approach to estimate the step length for healthy users and geriatric patients based on two foot-mounted IMU’s, one on each foot. The benefit of a deep learning approach is that no features need to be calculated as the deep learning algorithm will try to extract these features automatically. However, the drawback is that deep learning networks are computationally intensive during the training phase and that a very large data set is necessary to achieve good performance.

In [9,10], the authors use, respectively, only four and five self-chosen features that can be useful to estimate the step length. However, the authors do not verify if each of these features is indeed useful and if other features can be used to achieve better performance. Hence, in this paper, we evaluate a large set of features for step length estimation algorithms to select a subset of features to be used in the step length estimation process. As the complexity of both the training and the inference phase of the supervised learning algorithms increases with the number of used features, we cannot simply use this large feature set in our step length estimation algorithm so we need to select a subset. However, if we just reduce the number of features to lower the complexity, this might have a strong adversary effect on the performance of the algorithm. To the authors’ best knowledge, the optimal selection of features for step length estimation has not been considered yet. In this work, we evaluate the contribution of different features and propose a systematic approach to select the features. This approach resembles the approach used in our previous work [13], where feature selection was applied to step counting instead of step length estimation on the data of only one test subject. However, as step length estimation is a regression problem and step counting is a classification problem, this will have an impact on the feature selection algorithm. Hence, different metrics to rank the features, and metrics to evaluate the performance of the step length estimator needs to be used. Further, we also use data from multiple test persons so that we can now evaluate if the feature selection and step length estimation trained on certain test persons, also performs well on other test persons.

The rest of the paper is organized as follows. In Section 2, we introduce the set of features that are extracted from the measured acceleration. Then, in Section 3, we define two metrics to rank the features, which are used in Section 4 to select the subset of the extracted features that results in the best performance. In Section 5, we verify the performance of the selected features on multiple machine learning algorithms. Finally, conclusions will be given in Section 6.

## 2. Feature Extraction from the Accelerometer Signal

**Figure 1:**
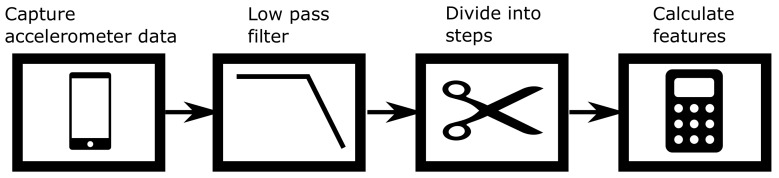
Process for feature extraction.

**Figure 2:**
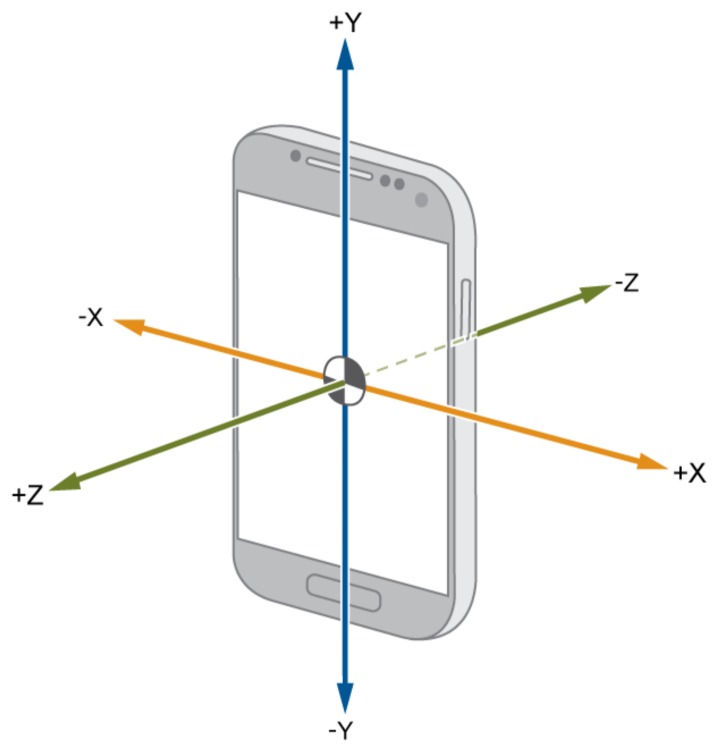
Coordinate system smartphone [13].

**Figure 3:**
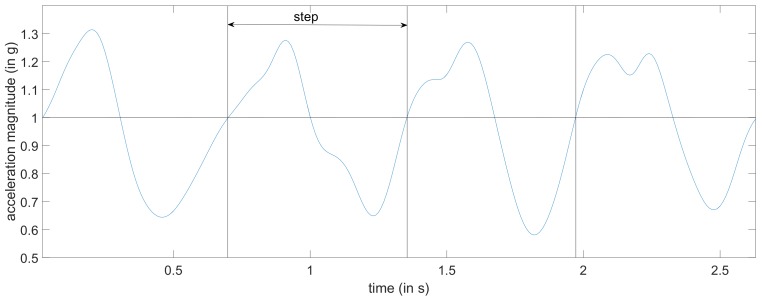
Example of the measured acceleration magnitude |a| with the boundaries for each step.

Before we can rank our features, we first need to extract them from the IMU data. The process to extract the different features from the data is given in Figure 1, which is very similar to the process in [13] except that in this paper the acceleration is divided into smaller fragments corresponding to one step, while in [13] the fragments could contain zero to five steps. We again restrict our attention to data captured by the accelerometer contained in a smartphone. The accelerometer outputs three signals (sampled at 100 Hz), corresponding to the acceleration in the *x*-, *y*-, and *z*-direction (see Figure 2). In this paper, we assume that the smartphone is always handheld in texting position iIn this paper we use the texting position as it is a logical choice if we want to navigate inside a building. However, other positions could be used as well but require additional training data) so that the *z*-axis is approximately aligned with gravity, i.e., the magnitude of the acceleration in the *z*-direction is larger than in the *x*- and *y*-direction. To smooth the measured acceleration sequences, we first applied a third-order low-pass Butterworth filter. Then, we determine the acceleration magnitude $\left|a\right|=\sqrt{a_{x}^{2}+a_{y}^{2}+a_{z}^{2}}$ of the filtered acceleration, where $a_{x}$, $a_{y}$ and $a_{z}$ are respectively the *x*-, *y*- and *z*-component of the filtered acceleration. Next, we divided the smoothed acceleration (the *x*, *y*, *z* and magnitude component) into smaller fragments that each correspond to one step. To this end, we implemented a step detection algorithm based on [14]. However, to ensure the quality of our dataset, we manually verified, and if necessary corrected, the results of the step detector. In Figure 3 we show an example of how the measured acceleration magnitude |a| from our handheld accelerometer is used to determine the boundaries for each step. To extract steps from the measured acceleration, we consider the instants where the acceleration magnitude |a| crosses 1 g, i.e., the gravitational force, with a positive slope as the beginning and/or endpoint of a step. These instants, where the acceleration crosses 1 g, approximately correspond to the instant at which the heel of one foot is lifted from the ground and to the instant at which the heel of the other foot strikes the ground, and hence can be seen as the begin and/or end of a step. Finally, we determine 128 features for each of the extracted steps. These features include, but are not limited to, the mean, variance, minimum and energy of the *x*-, *y*-, and *z*-acceleration components and the acceleration magnitude. In Appendix A, an overview of all features considered in this work are given.

To train and test our algorithm, we collected data corresponding to 837 steps from three different persons (two males and one female) with a handheld smartphone in texting position, where for each step, we manually determined the length of each step. The age of the three subjects varied from 23 to 59 years old, while their height was between 1.74 and 1.81 cm. In this work, the data set contains only data for steps that are extracted from a walking user. Our step length estimation algorithm, however, could be easily extended to other activities such as jogging and running by including additional data for these activities to our dataset. Only three different persons were used in this work, to test our algorithms. This allows us to make a first estimate of how the algorithm performs on data of unseen users. However, for more generalizable results, more test persons are required.

**Figure 4:**
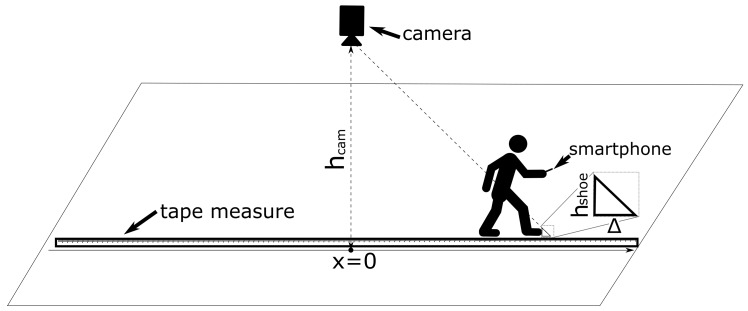
Method to determine ground truth for step lengths.

**Figure 5:**
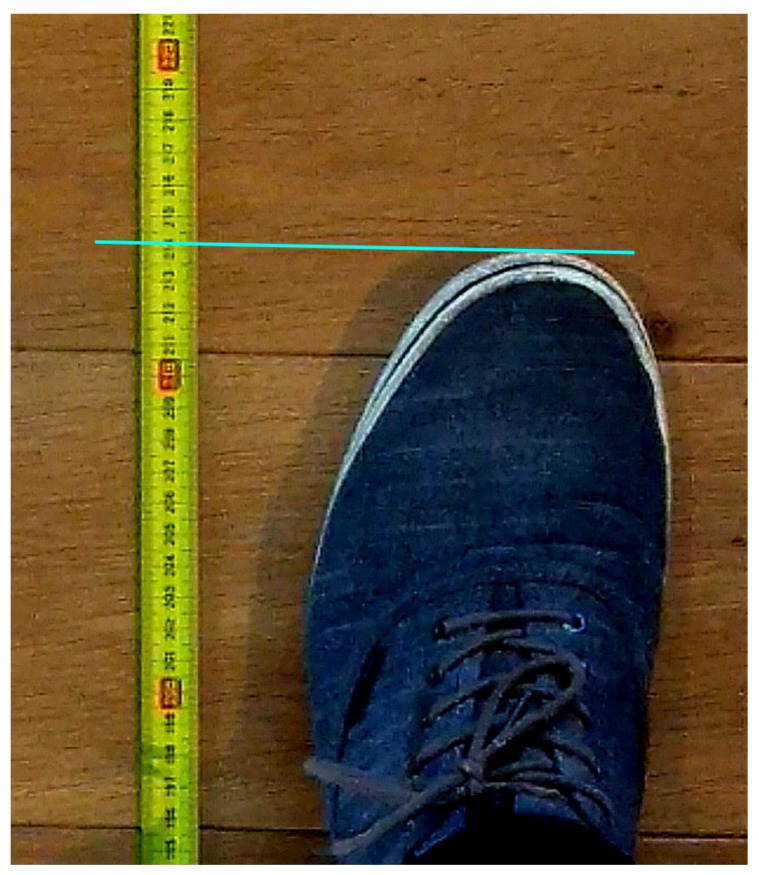
Example of how position of tip of foot is determined relative to tape measure from the camera image.

In this work, we use a single ceiling-mounted camera to obtain ground truth data of the step length for each step. In all our experiments, the user with a handheld smartphone, walked for approximately 5 m next to a tape measure so that based on the camera images, the step length can be determined accurately when the tip of a foot was stationary next to the tape measure. Our measurement setup to determine the ground truth is shown in Figure 4. When an occlusion occurred during a measurement, only the lengths of the steps before the occlusion are used in our dataset. In Figure 5, we show an example of how the position of the tip of a stationary foot is determined relative to the tape measure from the camera footage. We can see that although the numbers on the tape measure are not readable, we can still discern the tape measure markers every 0.5 cm. Hence, in this work, we round the estimated position of the tip of the foot to the nearest multiple of 0.5 cm. This approach resulted in step lengths between 37 and 101.5 cm. In Table 1, the properties of the collected data sets are shown. Figure 4 also shows that due to perspective errors, the position of the foot tip relative to the tape measure on the camera image differs from the true position by a distance of Δ. Using the properties of similar triangles, we can write that:

(1) $$\frac{h_{cam}}{h_{shoe}}=\frac{x+\Delta }{\Delta },$$

where $h_{cam}$ is the height of the camera, $h_{shoe}$ is the height of the tip of the shoe and *x* is the distance along the direction of the tape measure between the tip of the shoe and the point on the tape measure right below the camera. Note that Equation (1) is only valid for x>0, i.e., when the foot of the user is pointing away from the camera. For x<0, the perspective error Δ is equal to zero as in this case we do not suffer from perspective errors. Solving Equation (1) for Δ, we get that

$$\Delta \left(x\right)=\frac{h_{shoe}}{h_{cam}-h_{shoe}}\cdot x.$$

Hence, the perspective error grows when we move farther from the camera. This perspective error also induces an error $\Delta_{error}$ in the ground truth estimation of the step length $\Delta_{step}$. To find this error we need to subtract the perspective error at the end of the step, i.e., at $x=x^{\prime }+\Delta_{step}$, from the perspective error at the start of the step. Hence, the error on the estimation of the step length can be written as:

$$\Delta_{error}=\Delta \left(x^{\prime }+\Delta_{step}\right)-\Delta \left(x^{\prime }\right)=\frac{h_{shoe}}{h_{cam}-h_{shoe}}\cdot \Delta_{step},$$

which is independent of *x* and is only influenced by the height of the camera, the height of the tip of the shoe, and the step length. In this work, $h_{cam}$ was equal to roughly 260 cm and $h_{shoe}$ was approximately 4 cm. Hence, the error on the step length is equal to $\Delta_{error}=0.016\cdot \Delta_{step}$. In our experiments, the largest and the mean step length were respectively 101.5 cm and 69.9 cm, which results in a step length error $\Delta_{error}$ of 1.6 cm for the largest step and 1.1 cm for the mean step length. Hence, taking into the account the error from rounding the measured step length to the nearest multiple of 0.5 cm and the error $\Delta_{error}$ due to perspective, we can conclude that on average the error on the ground truth estimate of the step length is below 2 cm.

For each step in our data set, we determine the features given in Appendix A. These features are then arranged in a vector $x_{i}$, with *i* the step index and i∈[1,837]. Our algorithm, which we describe in the following sections, selects a subset of features that results in the best performance. We consider multiple supervised learning algorithms to test our feature selection method on. A problem, however, is that some of these learning algorithms require that the features are normalized to obtain good performance. Hence, to solve this problem we apply Z-score standardization to each feature, i.e., each feature is normalised so that the features in the training set have zero mean and unit standard deviation.

For new measurements, the features derived from these measurements will be normalized using the mean and standard deviation that was determined with the training set.

## 3. Feature Ranking

In a supervised-learning-based step length estimator, the algorithm must extract the length of a step from the obtained features. It is obvious that the choice of the features may have a large impact on the accuracy. For example, if a feature value is quasi-constant for different step lengths, it will not be able to discriminate between large and small steps, which makes it useless for step length estimation. To decide which of the features calculated in Section 2 are suitable for step length estimation, we consider two metrics, i.e., the correlation and mutual information, that express how much the step length influences a feature. The reason we consider these metrics is that they are frequently used in machine learning libraries to select the best features. For example, in this work we use the *scikit-learn* library, which allows us to select the best features based on either the F-score or the mutual information. To determine the F-score, first, the correlation needs to be calculated. As a result, we consider the correlation and mutual information to rank our features.

In the first metric, we first calculate the correlation $C_{j}$ between the *j*-th feature vector $X_{j}=\left[x_{1,j}^{\prime },x_{2,j}^{\prime },\ldots ,x_{N,j}^{\prime }\right]$ and the step length $y=[y_{1},y_{2},\ldots ,y_{N}]$ as:

$$C_{j}=\sum_{i=1}^{N}\frac{\left(x_{i,j}^{{}^{\prime }}-\mu_{x_{j}^{\prime }}\right)\left(y_{i}-\mu_{y}\right)}{\sigma_{x_{j}^{\prime }}\sigma_{y}},$$

where $N=|S_{1}\cup S_{3}\cup S_{4}{|}_{C}$ is the number of steps from experimental data sets $S_{1}$, $S_{3}$ and $S_{4}$ for which we know the step length, $x_{i,j}^{{}^{\prime }}$ is the *j*-th component of the normalised feature vector $x_{i}^{\prime }$ (the *j*-th feature) for the *i*-th step, and $\mu_{x_{j}^{\prime }}$ and $\sigma_{x_{j}^{\prime }}$ are the mean and standard deviation of the *j*-th normalized feature for the steps in our experimental data set. Further, $y_{i}$ is the length of the *i*-th step in our experimental data set, and $\mu_{y}$ and $\sigma_{y}$ are, respectively, the average and the standard deviation of the step length. The reason to use $S_{1}\cup S_{3}\cup S_{4}$ to rank the features is to make the ranking of the features less dependent on a specific user from our test data. We are interested in features with a high absolute value $|C_{j}|$ of the correlation, as they will be more likely to be useful for the step length estimation.

The second metric that we use to rank the features is the mutual information $I(x_{j},y)$ between the *j*-th feature $x_{j}$ and the corresponding step length *y*, and is defined by

$$I\left(x_{j},y\right)=\int \int p\left(x_{j},y\right)\log\left(\frac{p(x_{j},y)}{p\left(x_{j}\right)p\left(y\right)}\right)dx_{j}dy,$$

where $p(x_{j},y)$ is the joint probability function of the *j*-th feature and the step length, and $p(x_{j})$ and p(y) are the marginal distributions of respectively $x_{j}$ and *y*. As the joint and marginal probability functions are not known, the mutual information is approximated from the gathered data $X_{j}$ and y as described in [15,16]. The mutual information is a measure of the dependency between two variables, i.e., the feature value and the step length in our case. Taking into account that a large mutual information indicates that by observing the feature, the uncertainty on the step length will be reduced significantly, we are interested in features having a large mutual information.

**Figure 6:**
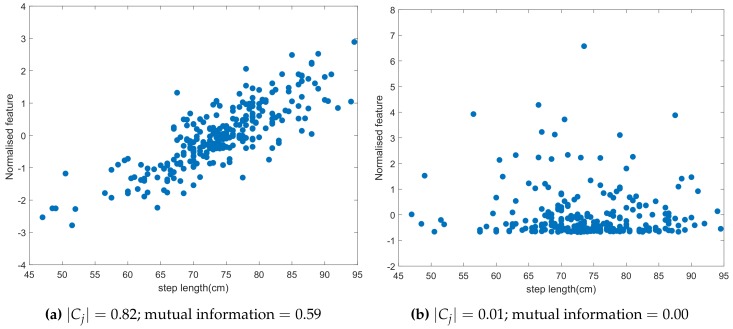
Feature values in function of the step length for (**a**) a good feature (**b**) a bad feature.

For each of the 128 features, we determined both metrics. An example is illustrated in Figure 6 for two features, i.e., a feature with a high correlation and mutual information (a good feature), and a feature with a low correlation and mutual information (a bad feature). As can be observed, the feature values for the good feature increase with an increasing step length, while for the bad feature, no clear trend is visible. Hence, the bad feature Figure 6b will be less suited to estimate the step length. The feature selection algorithm described in the next section needs as input a ranked set of features. For both metrics, features with a high value of the metric will be more suited to determine the step length. Hence, for each of both metrics, we generate a ranked feature set by sorting the features in decreasing order of the considered metric (correlation or mutual information).

## 4. Feature Selection Method

In the previous section, we introduced two metrics to rank the features, resulting in the ranked sets $\Omega_{r,C}$ for the correlation metric and $\Omega_{r,\mathrm{MI}}$ for the mutual information metric. In this section, we describe an algorithm that starts from one of the ranked sets $\Omega_{r,i}$, i={C,MI}, to select a subset $\Omega_{f,i}$ of features that results in optimal performance with a limited number of features. Considering that this selection algorithm can be used irrespective of the used ranking approach, we drop the index *i* in this section to simplify notations. To decrease the necessary computation time to find the best feature set, we limit the ranked feature set $\Omega_{r}$ to the *K* features with the highest rank, where *K* is a parameter that can be used to trade off performance for computation time. A higher value for *K* improves the performance but also results in a longer computation time, while a lower value deteriorates performance and reduces computation time. To select the best features, we use a similar approach as in [13], but the main difference is that for the step length estimation problem we need to estimate a continuous variable, while [13] considered a discrete output. This requires that we need to use different ranking and accuracy metrics, and machine learning algorithms in our feature selection algorithm.

In this feature selection algorithm, we will sequentially update the final feature set $\Omega_{f}$. To find the best feature set, we use a part of our experimental data from Table 1, which we will call $S_{\mathrm{fs}}$. To evaluate the performance of the selected feature subset $\Omega_{f}$, we randomly select 80% of $S_{\mathrm{fs}}$ as the training set $S_{\mathrm{fs},\mathrm{train}}$ to train the supervised algorithm, while the remainder is used as the test set $S_{\mathrm{fs},\mathrm{test}}$ to test the model derived from $S_{\mathrm{fs},\mathrm{train}}$. To minimize the influence of the selected training set, we repeat the training and testing of $\Omega_{f}$ 100 times using different randomly selected training sets. In this paper, we use the mean absolute error $mae_{f}$ of the step length estimation to evaluate the overall performance, though other performance measures could also be used in our algorithm, e.g., the root-mean-square error or the relative absolute error. The overall performance of our step length estimator is found by averaging the mean absolute error on the test set over the 100 runs, where the mean absolute error is given by

$$mae_{f}=\sum_{i=1}^{N^{\prime }}\frac{|y_{pred}\left(i\right)-y_{test}\left(i\right)|}{N^{\prime }},$$

with $N^{\prime }={\left|S_{\mathrm{fs},\mathrm{test}}\right|}_{C}$ the cardinality of the test set $S_{\mathrm{fs},\mathrm{test}}$, $y_{pred}\left(i\right)$ the predicted step length of the *i*-thstep, and $y_{test}\left(i\right)$ the true length of that step. By definition $mae_{f}\geq 0$. Our algorithm searches for the features that minimize the $mae_{f}$. To this end, the algorithm updates the final feature set $\Omega_{f}$ in three phases, i.e., the initialization phase, the addition phase and the deletion phase.

**Figure 7:**
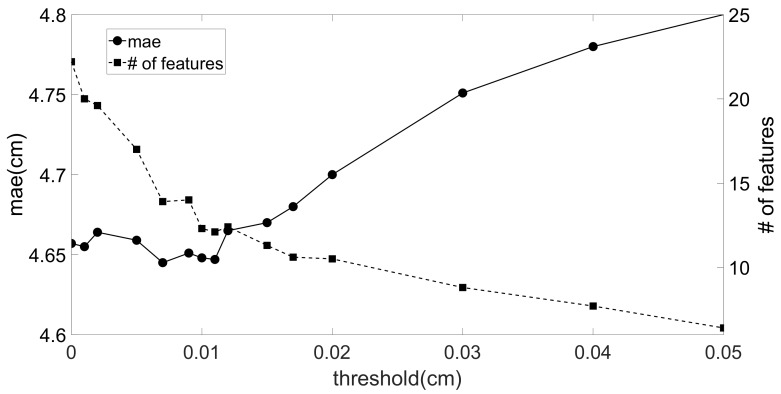
Mae and number of features as a function of the threshold in the addition phase.

1. Initialisation phase: as a first step in our algorithm, we determine the correlation or mutual information between a feature and the step length so that we can rank the features according to the chosen metric (Section 3). This results in the ranked feature set $\Omega_{r}$. Next, we define the final feature set $\Omega_{f}$, which, at first, contains only the feature with the highest score in the ranked set, which is simultaneously removed from $\Omega_{r}$. This feature set $\Omega_{f}$ is then used to train the supervised learning algorithm on $S_{\mathrm{fs},\mathrm{train}}$ after which the trained model is used to determine the mean absolute error $mae_{f}$ on the test set $S_{\mathrm{fs},\mathrm{test}}$.
2. Addition phase: in the addition phase, we one by one test each feature in $\Omega_{r}$. If a feature results in a significantly lower (better) mean absolute error, i.e., if $mae_{temp}<mae_{f}-threshold$, this feature is added to $\Omega_{f}$, where the mean absolute error $mae_{temp}$ on the temporary feature set is determined by training the learning algorithm on the temporary feature set
  $$\Omega_{temp}=\Omega_{f}\cup \left\{x_{new}\right\},$$
  with $x_{new}$ the new feature that is evaluated. The threshold is used to prevent that features with a very limited impact on the performance becomes part of the final feature set, which can result in a final feature set that is overtrained on our steps in $S_{\mathrm{fs},\mathrm{test}}$. This implies that it will perform well for this set but can have a worse performance for feature sets derived from new steps. Besides preventing overtraining, with this threshold, we can also reduce the training time. In Figure 7 we show the average mae, i.e., the performance of our feature selection algorithm, and the average number of features in the final feature set as a function of the threshold in the addition phase. The average is found by averaging over the five used supervised learning algorithms, i.e., k-nearest neighbors, RBF SVM regression, decision tree, elastic net, and ridge regression. From this figure, we observe that a good choice for the threshold is around 0.01 cm. Lower thresholds resulted in larger feature sets with practically the same performance, while higher thresholds resulted in performance degradation. Therefore, we use 0.01 cm for this threshold in our paper.
  We distinguish two cases for $x_{new}$:
  - In the previous step the temporary feature $x_{new}$ did result in $mae_{temp}<mae_{f}-threshold$. In this case:
    (2) $$mae_{f}\leftarrow mae_{temp}$$
    (3) $$\Omega_{f}\leftarrow \Omega_{temp}$$
    (4) $$\Omega_{r}\leftarrow \Omega_{r}\backslash \{x_{new}\}.$$
    As a next step, we again start with testing the feature of $\Omega_{r}$ with the highest rank that is not in $\Omega_{f}$. The reason for this look-back phase is that a feature that was not added to the final set in an earlier stage, for example because it is highly correlated with one or more features that are already present in $\Omega_{f}$ and therefore did not have noticeable influence on the performance, can result in a higher performance in combination with a feature that has a lower rank. During our tests, we found that 26.0% of the features that were added to the final set were not added the first time they were considered in the addition phase.
  - The tested feature did not result in a better performance and hence, is not added to $\Omega_{f}$. In this case, we continue with the look-forward phase, i.e., we test the next feature in $\Omega_{r}$ that has a lower rank than the previously tested feature.
  When a feature $x_{new}$ is added to $\Omega_{f}$, the feature is also removed from $\Omega_{r}$.
3. Deletion phase: in the previous phase, i.e., the addition phase, we tested all the features in the ranked feature set and included every feature that resulted in a lower $mae_{f}$. After this phase, we now test if eliminating a feature from $\Omega_{f}$ has a significant influence on the performance. To do so, we set
  $$\Omega_{temp}=\Omega_{f}\backslash \left\{x_{del}\right\},$$
  where $x_{del}$ is the feature that was added first to the final set, and determine the mean absolute error $mae_{temp}$ when $\Omega_{temp}$ is used to train the machine learning algorithm.
  This again results in two possible cases:
  - $mae_{temp}>mae_{f}$, which means that when $x_{del}$ is removed from $\Omega_{f}$ the performance degrades and hence, this feature must be kept. In the next iteration of our algorithm, we set $x_{del}$ equal to the next feature in $\Omega_{f}$
  - $mae_{temp}\leq mae_{f}$: this means that
    (5) $$mae_{f}\leftarrow mae_{temp}$$
    (6) $$\Omega_{f}\leftarrow \Omega_{temp}$$
    (7) $$\Omega_{r}\leftarrow \Omega_{r}\cup \left\{x_{del}\right\}$$
    $x_{del}$ is removed from $\Omega_{f}$, $x_{del}$ is again added to ranked feature set $\Omega_{r}$, and as the next $x_{del}$ we again use the feature of the final feature set $\Omega_{f}$ we added the longest ago (look-back phase). The reason why the performance can increase when a feature is removed from $\Omega_{f}$ is that the combined information of one or more features of $\Omega_{f}$ contain similar information and hence, this feature can be deleted. In our experiments, 40.9% of the features are again removed from the final feature set after that they were added.
  Note that in the deletion phase, we do not use a threshold in contrast to the addition phase as this did not result in a better performance. The deletion phase ends when the last feature in $\Omega_{f}$ is evaluated and not removed from $\Omega_{f}$.

When at least one feature was removed from $\Omega_{f}$, we go through an extra addition and deletion phase until no features are removed from the final feature set. At this point, we stop our feature selection algorithm as it now yields optimal performance in the sense that adding another feature from $\Omega_{r}$ or removing a feature from $\Omega_{f}$ will not improve the accuracy further. From our experiments, it followed that only 1.9% of the features were not added during the first addition phase. Hence, typically our feature selection algorithm will go through two addition and deletion phases. In the next paragraph, we illustrate our feature selection algorithm on a radial basis function support vector machine (RBF SVM) with regression, where we only look at the 50 features with the highest rank (K=50). To train and test our feature selection algorithm, we set $S_{\mathrm{fs}}=S_{1}\cup S_{3}$ (see Table 1). Table 2 contains the union $\Omega_{f,C}\cup \Omega_{f,\mathrm{MI}}$ of the features selected by the feature selection algorithm, where $\Omega_{f,C}$($\Omega_{f,\mathrm{MI}})$ is the final feature set when the correlation (mutual information) is used to rank the features. Looking at Table 2, we can see that approximately half of the features, i.e., features 1–11, are extracted from the time-domain signal, while the other features are derived from the frequency domain signal. We also notice that a large part of the features are obtained from the *z*-component or the magnitude of the acceleration. This can be explained by the approximately horizontal handheld smartphone combined with the up-and-down movement due to a step that is mainly present in the *z*-component of the acceleration.

In Table 3, we compare the final feature set for the two ranking approaches, i.e., correlation and mutual information. Note that both ranking approaches result in a similar number of features in the final sets $\Omega_{f,i}$, i∈{C,MI}. The table also reveals there is a large overlap between the final features for both approaches. In total 12 features (out of the 21 in Table 2) appear in both sets. Hence, we can conclude that our algorithm is able to find similar feature sets for different ranking approaches.

## 5. Results and Validation

In this section, we compare the performance of machine-learning-based step length estimators employing the proposed systematic feature selection algorithm. We apply the selection algorithm to five supervised-learning algorithms, being k-nearest neighbors, RBF SVM regression, decision tree, elastic net and ridge regression. First, we utilize $S_{\mathrm{fs}}=S_{1}\cup S_{3}$ from Table 1 to determine the best features using the selection algorithm from Section 4 and the two metrics of Section 3, and we also determine the performance of our step length estimation algorithm on the same data. Additionally, we compare this performance with state-of-the-art step length estimators. For this comparison, we train and test these algorithms on the same dataset of our own step length estimation algorithm. Next, we validate the performance of our algorithm (trained on the data set $S_{1}\cup S_{3}$) on new data (data set $S_{2}$) from test person 1, who already provided data for the training of our algorithm. Finally, we will also validate our algorithm (trained on the data set $S_{1}\cup S_{3}$) on data from a third, new test person (data set $S_{4}$).

In Table 4, we show the mean absolute error of our step length estimator with different supervised learning algorithms and both ranking approaches trained on the feature set that results from the feature selection algorithm in Section 4 (trained and tested on $S_{\mathrm{fs}}=S_{1}\cup S_{3}$). In the same table, we also show the mean absolute error of two state-of-the-art methods [9,10] that use a fixed feature set on a neural network. As mentioned in Section 1, ref. [9] considers a fixed set of four features which are the maximum, the minimum, the variance and the integral of the acceleration. Ref. [10] on the other hand uses a set of five features which are the maximum, standard deviation and mean of the acceleration, the stride frequency and the height of the user. For a fair comparison, we obtain this mean absolute error from the same 100 training/test sets that were used in the feature selection algorithm. In Table 4, we see that the difference between all considered algorithms is rather small. From this table, it follows that the k-nearest neighbors algorithm has the lowest mean absolute error for both feature ranking approaches, i.e., $mae_{\mathrm{kNN},C}=4.24$ cm and $mae_{\mathrm{kNN},\mathrm{MI}}=4.19$ cm. The decision tree algorithm, on the other hand, has the highest mean absolute error for both approaches, i.e., 4.94 cm and 4.96 cm for the correlation and mutual information metrics, respectively. In general, we notice that both approaches result in similar performances for all supervised learning algorithms. If we compare the results of our step length algorithm with the results of the neural network algorithms from [9,10], we notice that only for the decision tree algorithm, the performance of our algorithm is worse than [10], while for all other combinations our procedure performs as well or better than both neural network approaches.

Next, we will determine if the optimal feature set derived from $S_{\mathrm{fs}}=S_{1}\cup S_{3}$ also performs well on new data (data set $S_{2}$) from a test person that is already used to train the algorithm. To this end, we determine the optimal features for each machine learning algorithm and ranking approach and train the machine learning algorithm with the resulting features on $S_{1}\cup S_{3}$. Afterwards, we tested the trained machine learning model on the examples of data set $S_{2}$ to validate our algorithm on new data. In Table 5, we show the results from these tests. Additionally, we now also compare our approach to three parametric step length estimators [3,4,5]. To obtain these results, we use the data from $S_{\mathrm{fs}}$ to find the optimal values for the parameters of these approaches and use these optimal values to evaluate the models on $S_{2}$. We again note that in general our step length estimator performs better than the methods from [9,10]. Only when we use the decision tree algorithm, the performance is worse. In contrast to the results from Table 4, now the RBF SVM regression algorithm has the best performance instead of the k-nearest neighbors algorithm. We also note that [3] is the parametric approach that results in the best performance, which is comparable with the performance of [9]. The other parametric models result in a significantly lower performance.

Finally, we also validate our step length estimation algorithm on data from a different test person than the test persons used for the training data. For this test, we again train our step length estimation on the data from $S_{1}\cup S_{3}$ using the optimal features for this data set. To validate the performance of our algorithm we use data from a third test person, i.e., data set $S_{4}$ in Table 1. In a similar way as in the previous paragraph, we again compare our approach with the three parametric models. The results are also shown in Table 5. The decision tree algorithm again has the worst performance, and again our step length algorithm outperforms [9,10]. For the parametric approaches, we again observe that [3] results in the best parametric model but still with, in general, a worse performance than our step length estimators. While the results for validation set $S_{2}$ suggest that the RBF SVM algorithm is more robust to new data, it follows from the results for validation set $S_{4}$ that our feature selection approach combined with the RBF SVM regression does not result in the best performance when tested on data from a new test person. Comparing the results from validation set $S_{2}$ and $S_{4}$, it follows that ridge regression and elastic net were the most robust to new data and achieve a better performance than [9,10].

Until now, we determined the optimal feature set and trained our step length estimator with data from the set $S_{1}\cup S_{3}$, containing data from two test persons. Now, we will evaluate to what extent the performance of the step length estimator changes when data from only one test person is used to train the step length estimator. Additionally, we also determine the leave-one-subject-out (LOSO) cross-validated results, which can also be derived from these simulations. In Table 6, Table 7 and Table 8, we show the results when the step length estimator is trained with data from test persons X and/or Y, and validated with data from test person Z. First, weobserve that when the algorithm is trained with data from test person X or Y only, the validation performance for data from test person Z may differ significantly. A reason for this observation might be that the steps of test person Z are more similar to the steps of e.g., test person X, resulting in a better performance than when the algorithm is trained on test person Y. Further, when the estimator is trained on the data from both test persons X and Y (i.e., by the union of the data sets), the resulting mae is almost always contained in the interval between the mae’s obtained with the single test sets only. Hence, if we can determine for each new person to which of the test persons in the training data it corresponds the most, we can improve the performance of the step length estimator. On the other hand, choosing the wrong test person for training our algorithm deteriorates the performance. A difficulty with this approach, however, is that we need to obtain a trained model for each new user depending on the most similar test person in the training data. A solution for this is to store a trained model for each test person in the training data. This would, however, require a large storage for a training set with a large number of different users. In Table 9, we give the LOSO cross validated result of our step length estimator. These results can be extracted from Table 6, Table 7 and Table 8 by averaging over the columns $S_{3}\cup S_{4}$, $S_{1}\cup S_{4}$ and $S_{1}\cup S_{3}$ respectively. From this table, we can see that with a LOSO cross validated mean absolute error of 5.17 cm and 5.11 cm for respectively the correlation and mutual information approach, the ridge regression algorithm results in the best performance.

## 6. Conclusions

In this paper, we used the correlation and mutual information to identify and rank the features that could potentially be used for step length estimation and proposed a method to systematically build a feature set for a machine-learning-based step length estimator. We currently use data of three test persons, which allows us to make a first estimate of the performance of our algorithm. However, in future work, we will evaluate our algorithm on more test persons to obtain more generalizable results. We trained multiple machine learning algorithms with their selected feature set and compared the different algorithms in terms of mean absolute error. To verify the robustness of our algorithm, we validated our machine learning approach on data from a different test person than the ones used for training our algorithm. The ridge regression algorithm combined with the feature ranking approach based on the mutual information resulted in a mean absolute error of 3.48 cm when tested on a test person that was used for training the algorithm, and a mean absolute error of 4.19 cm when tested on a new test person. We also compared our method with two neural network-based step length estimators and both algorithms achieved a worse performance than the presented algorithm. We showed that our step length estimator can make an accurate step length estimate, even for new test persons, without changing the algorithm, and that we achieve a similar or better accuracy than current state-of-the-art methods. Finally, we also found that for a new user the performance can be improved, if we can determine which of the test persons in the training data corresponds the most to the new person. To allow other researchers to compare our models against their solution, a test script is provided (see the Supplementary Materials) to evaluate our models on new step data.

## Figures and Tables

**Table 1:** Properties of gathered steps and corresponding step lengths.

Data Set	Test Person	Steps	Minimum Step Length (cm)	Maximum Step Length (cm)	Mean Step Length (cm)
$S_{1}$	1	257	47	94.5	74.1
$S_{2}$	1	135	53.5	101.5	70.5
$S_{3}$	2	219	37	100.5	66.7
$S_{4}$	3	226	43.5	88.5	68.3
	total	837	37	101.5	69.9

**Table 2:** Features in $\Omega_{f,C}\cup \Omega_{f,\mathrm{MI}}$ for the RBF SVM algorithm, where $\Omega_{f,i}$ is the final feature set for ranking approach i∈{C,MI}. Features marked with an * are features that are part of both feature sets.

Feature Number	Feature Description
1	Standard deviation of |***a***|
2 *	Minimum of *a*_*y*_
3 *	Minimum of *a*_*z*_
4	Minimum of |***a***|
5	Mean absolute deviation of *a*_*z*_
6	Interquartile range of *a*_*z*_
7	Interquartile range of |***a***|
8 *	Energy of *a*_*y*_
9 *	Signal magnitude area of acceleration
10 *	Mean of |**a**|
11	Number of minima in |***a***|
12 *	Maximum of FFT of *a*_*z*_
13 *	Maximum of FFT of |***a***|
14	Standard deviation of FFT of *a*_*y*_
15 *	Standard deviation of FFT of *a*_*z*_
16 *	Mean absolute deviation of FFT of *a*_*y*_
17	Mean absolute deviation of FFT of |***a***|
18 *	Energy of FFT of *a*_*y*_
19 *	Energy of FFT of |***a***|
20	Energy of FFT of *a*_*y*_ between 1 and 5 Hz
21 *	Energy of FFT of |***a***| between 1 and 5 Hz

**Table 3:** Final feature set $\Omega_{f}$ for the two ranking approaches.

$\Omega_{f,C}$	2	3	7	8	9	10	11	12	13	14	15	16	18	19	21			
$\Omega_{f,\mathrm{MI}}$	1	2	3	4	5	6	8	9	10	12	13	15	16	17	18	19	20	21

**Table 4:** Accuracy comparison of different machine learning algorithms and approaches with two neural network step length algorithms.

Mean Absolute Error	Correlation Approach (cm)	Mutual Information Approach (cm)
k-nearest neighbors	4.24	4.19
RBF SVM regression	4.54	4.56
Decision tree	4.94	4.96
Elastic net	4.71	4.78
Ridge regression	4.77	4.79
[9]	5.03	
[10]	4.77	

**Table 5:** Accuracy comparison of different machine learning algorithms and approaches with two neural network step length algorithms and three parametric approaches, validated on new data of a test person that was used for training and new data of a new test person.

Mean Absolute Error	Validation on Person in Training Data ($S_{2}$)		Validation on New Person ($S_{4}$)	
	$\begin{array}{c} \mathrm{Correlation}\;\mathrm{Approach}\\ \left(\mathrm{cm}\right) \end{array}$	$\begin{array}{c} \mathrm{Mutual}\;\mathrm{Information}\;\mathrm{Approach}\\ \left(\mathrm{cm}\right) \end{array}$	$\begin{array}{c} \mathrm{Correlation}\;\mathrm{Approach}\\ \left(\mathrm{cm}\right) \end{array}$	$\begin{array}{c} \mathrm{Mutual}\;\mathrm{Information}\;\mathrm{Approach}\\ \left(\mathrm{cm}\right) \end{array}$
k−nearestneighbours	3.80	3.65	5.21	5.22
RBFSVMregression	3.56	3.39	4.53	4.85
Decisiontree	4.90	4.11	5.60	5.80
Elastic net	3.58	3.53	4.32	4.16
Ridge regression	3.54	3.48	4.34	4.19
[9]	4.94		6.27	
[10]	4.26		8.22	
[3]	4.89		5.73	
[4]	7.57		6.19	
[5]	6.59		6.21	

**Table 6:** Accuracy comparison of different training sets for the different machine learning algorithms validated on $S_{1}$.

Mean Absolute Error	$\begin{array}{c} \mathrm{Correlation}\mathrm{Approach} \end{array}$ (cm)			$\begin{array}{c} \mathrm{Mutual}\mathrm{Information}\mathrm{Approach} \end{array}$ (cm)		
Trainingset	$S_{3}$	$S_{4}$	$S_{3}\cup S_{4}$	$S_{3}$	$S_{4}$	$S_{3}\cup S_{4}$
k−nearestneighbours	5.83	6.28	6.09	6.05	7.10	6.71
RBFSVMregression	5.37	6.35	5.80	5.20	6.33	5.88
Decisiontree	6.37	7.57	6.24	6.44	7.59	6.54
Elastic net	5.38	6.07	6.16	6.01	6.11	6.39
Ridge regression	6.08	6.29	5.55	5.92	6.19	5.47

**Table 7:** Accuracy comparison of different training sets for the different machine learning algorithms validated on $S_{3}$.

Mean Absolute Error	$\begin{array}{c} \mathrm{Correlation}\mathrm{Approach} \end{array}$ (cm)			$\begin{array}{c} \mathrm{Mutual}\mathrm{Information}\mathrm{Approach} \end{array}$ (cm)		
Trainingset	$S_{1}$	$S_{4}$	$S_{1}\cup S_{4}$	$S_{1}$	$S_{4}$	$S_{1}\cup S_{4}$
k−nearestneighbours	6.59	5.84	6.11	7.92	5.86	5.93
RBFSVMregression	8.78	5.04	5.80	8.25	5.02	5.86
Decisiontree	7.50	6.30	5.93	7.02	6.14	6.49
Elastic net	9.49	5.24	5.72	8.31	5.12	5.77
Ridge regression	8.03	5.16	5.62	8.08	5.25	5.66

**Table 8:** Accuracy comparison of different training sets for the different machine learning algorithms validated on $S_{4}$.

Mean Absolute Error	$\begin{array}{c} \mathrm{Correlation}\mathrm{Approach} \end{array}$ (cm)			$\begin{array}{c} \mathrm{Mutual}\mathrm{Information}\mathrm{Approach} \end{array}$ (cm)		
Trainingset	$S_{1}$	$S_{3}$	$S_{1}\cup S_{3}$	$S_{1}$	$S_{3}$	$S_{1}\cup S_{3}$
k−nearestneighbours	5.71	3.71	5.21	6.15	3.80	5.22
RBFSVMregression	5.99	3.23	4.53	5.65	3.26	4.85
Decisiontree	7.35	4.37	5.60	7.85	4.91	5.80
Elastic net	6.02	3.34	4.32	5.40	3.42	4.16
Ridge regression	5.56	3.27	4.34	5.65	3.27	4.19

**Table 9:** Leave-one-subject-out (LOSO) cross validated mean absolute error for our step length estimation algorithm.

Mean Absolute Error	Correlation Approach (cm)	Mutual Information Approach (cm)
k-nearest neighbours	5.80	5.95
RBFSVMregression	5.38	5.53
Decisiontree	5.92	6.28
Elastic net	5.40	5.44
Ridge regression	5.17	5.11

## Supplementary Materials

The following are available online at https://www.mdpi.com/1424-8220/20/3/778/s1.

## Appendix A

In this appendix, we give all the features that are used in our feature selection algorithm. In total we consider 128 features derived from the acceleration samples in one step. 33 features are derived from the time domain, 85 from the frequency, and an additional 10 features are derived from the set of peaks/troughs in the acceleration signal of one step. In Table A1, we overview all calculated features. In each cell of the table, we indicate if the feature is determined for the acceleration in the *x*-, *y*- and *z*-axis, and the magnitude of the acceleration ($\left|□\right|$). e.g., $x,y,z,\left|□\right|$ means that the feature is determined for the *x*, *y* and *z* components of the acceleration and for the magnitude as well. A ${}^{\prime }{+}^{\prime }$ sign indicates that several components of the acceleration are used to determine one feature. e.g., the *x*, *y* and *z* components of the acceleration are used to determine the signal magnitude area.

**Table A1:** Considered features in this work.

	Time Domain	Frequency Domain	Peaks/Troughs
**Mean**	$x,y,z,\left|□\right|$	$x,y,z,\left|□\right|$	$\left|□\right|$
**Standard deviation**	$x,y,z,\left|□\right|$	$x,y,z,\left|□\right|$	$\left|□\right|$
**Minimum**	$x,y,z,\left|□\right|$	$x,y,z,\left|□\right|$	
**Maximum**	$x,y,z,\left|□\right|$	$x,y,z,\left|□\right|$	
**Mean absolute deviation**	$x,y,z,\left|□\right|$	$x,y,z,\left|□\right|$	
**Interquartile range**	$x,y,z,\left|□\right|$	$x,y,z,\left|□\right|$	$\left|□\right|$
**Energy**	$x,y,z,\left|□\right|$	$x,y,z,\left|□\right|$	
**Signal magnitude area**	x+y+z	x+y+z	
**Correlation between** ***x*** **and** ***y*** **acceleration**	x+y		
**Correlation between** ***x*** **and** ***z*** **acceleration**	x+z		
**Correlation between** ***y*** **and** ***z*** **acceleration**	y+z		
**Number of samples above 1.1 g**	$\left|□\right|$		
**Frequency at maximum of FFT**		$x,y,z,\left|□\right|$	
**Weighted average**		$x,y,z,\left|□\right|$	
**Kurtosis**		$x,y,z,\left|□\right|$	
**Skewness**		$x,y,z,\left|□\right|$	
**Energy: 0 to 5 Hz**		$x,y,z,\left|□\right|$	
**Energy: 5 to 10 Hz**		$x,y,z,\left|□\right|$	
**Energy: 10 to 15 Hz**		$x,y,z,\left|□\right|$	
**Energy: 15 to 20 Hz**		$x,y,z,\left|□\right|$	
**Energy: 20 to 25 Hz**		$x,y,z,\left|□\right|$	
**Energy: 25 to 30 Hz**		$x,y,z,\left|□\right|$	
**Energy: 30 to 35 Hz**		$x,y,z,\left|□\right|$	
**Energy: 35 to 40 Hz**		$x,y,z,\left|□\right|$	
**Energy: 40 to 45 Hz**		$x,y,z,\left|□\right|$	
**Energy: 45 to 50 Hz**		$x,y,z,\left|□\right|$	
**Number of peaks/troughs**			$\left|□\right|$
**Mean time between peaks/troughs**			$\left|□\right|$
**Number of features**	33	85	10
